# Structure of helminth communities of rodents in three areas of the Atlantic Forest, Paraná state, Brazil

**DOI:** 10.1590/S1984-29612026003

**Published:** 2026-04-20

**Authors:** Andressa Santos de Carvalho da Silva, Beatriz Elise de Andrade Silva, Camila dos Santos Lucio, Fernanda de Sousa Pacheco, Bernardo Rodrigues Teixeira, Rosana Gentile

**Affiliations:** 1 Fundação Oswaldo Cruz, Laboratório de Biologia e Parasitologia de Mamíferos Silvestres Reservatórios, Rio de Janeiro, RJ, Brasil; 2 Fundação Oswaldo Cruz, Programa de Pós-graduação em Biodiversidade e Saúde, Rio de Janeiro, RJ, Brasil; 3 Universidade do Estado do Rio de Janeiro, Departamento de Microbiologia, Imunologia e Parasitologia, Laboratório de Helmintologia Romero Lascasas Porto, Rio de Janeiro, RJ, Brasil

**Keywords:** Cestoda, nematoda, parasitism, rodentia, Cestoda, nematoda, parasitismo, rodentia

## Abstract

Parasites play a key role in the structure of ecosystems; however, there is still a large gap in the knowledge of the diversity of parasites of wild hosts. The objective of this study was to describe the structure of rodent helminth communities in three municipalities in the state of Paraná, Brazil. A total of 66 infected hosts of seven rodent species were detected as follows (analysed/infected): *Akodon montensis* (57/33), *Akodon paranaensis* (16/5), *Euryoryzomys russatus* (6/4), *Mus musculus* (42/10), *Oligoryzomys nigripes* (16/11), *Rattus rattus* (1/1) and *Thaptomys nigrita* (2/2). From the phylum Nematoda, we found twenty-six morphotypes, among which thirteen were identified at the species level. We identified three morphotypes of the phylum Platyhelminthes. No significant differences were found between the species richness and total abundance of helminths in relation to the sex or age of the hosts for *A. paranaensis, O. nigripes* or *M. musculus*. In *A. montensis,* young individuals presented a greater abundance than did adults. Here, we report for the first time *Syphacia carlitosi* and *Syphacia evaginata* and the genus *Pterygodermatites* in the rodent *O. nigripes.* This is also the first report of *Heterakis spumosa* in *A. montensis* and *A. paranaensis*, and the first report of *Stilestrongylus eta* in *M. musculus* and *E. russatus.*

## Resumo

Os parasitos possuem um papel fundamental na estrutura dos ecossistemas, contudo, ainda há uma extensa lacuna sobre o conhecimento da diversidade de parasitos de hospedeiros silvestres. O objetivo deste trabalho foi descrever a estrutura das comunidades de helmintos de roedores em três municípios do estado do Paraná, Brasil. Foram coletados 66 hospedeiros infectados das espécies *Akodon montensis* (57/33), *Akodon paranaensis* (16/5), *Euryoryzomys russatus* (6/4), *Mus musculus* (42/10), *Oligoryzomys nigripes* (16/11), *Rattus rattus* (1/1) e *Thaptomys nigrita* (2/2). Do filo Nematoda, encontramos 26 morfotipos, dentre os quais, treze foram identificados em nível de espécie. Do filo Platyhelminthes, encontramos três morfotipos*.* Não foram encontradas diferenças significativas entre a riqueza e abundância total de helmintos em relação ao sexo e idade dos hospedeiros para *A. paranaenses, O. nigripes* e *M. musculus*. Em *A. montensis,* indivíduos jovens tiveram maior abundância do que adultos. Aqui nós reportamos pela primeira vez as espécies *Syphacia carlitosi* e *Syphacia evaginata* e do gênero *Pterygodermatites* no reodor *O. nigripes.* Este é também o primeiro relato de *Heterakis spumosa* em *A. montensis* e *A. paranaensis*, e o primeiro relato da espécie *Stilestrongylus eta* em *M. musculus* e em *E. russatus*.

## Introduction

Knowledge of the diversity of parasites and how their communities are structured in their wild hosts are essential issues for species conservation and are indicators of ecosystem health (Gentile & D'Andrea, 2016). In addition, studies on parasite distributions in host populations and the factors that influence their occurrence at different scales contribute to the understanding of ecosystem dynamics (Lymbery, 2005; Krasnov et al., 2006; Johnson et al., 2013).

Urban growth and anthropogenic activities have had major environmental impacts on Brazilian biomes. These impacts cause a reduction in the natural habitats of the species, altering their composition and abundance. Thus, the advance of urban areas and agricultural and livestock activities over forested areas has facilitated the encounter of wild animals with human populations and domestic and farm animals; this type of contact allows infectious and parasitic agents to find new hosts and environments (Corrêa & Passos, 2001) contributing to the emergence or re-emergence of zoonotic diseases (Kruse et al., 2004).

The order Rodentia is the most diverse among mammals in the world (Burgin et al., 2018), representing more than 42% of the species and 39% of the genera (Patton et al., 2015). The study of rodents is important for public health because they can act as reservoirs of several parasites, such as Orthohantavirus, *Trypanosoma cruzi* and *Schistosoma mansoni* (Corrêa & Passos, 2001; Maldonado et al., 2006; Oliveira et al., 2014).

Among several groups of parasites, helminths play important roles in ecosystem maintenance and are widely used as indicators of environmental change (Gardner & Campbell, 1992; Lymbery, 2005). They are excellent models for investigating environmental changes across time and space because, as endoparasites, their abundance can be readily quantified.

The impact of parasitism on hosts and their populations can significantly alter their population structure (Marcogliese, 2005). Studies on rodent helminth communities have been conducted in Brazil, mainly in the states of Rio de Janeiro, Bahia, Minas Gerais and Santa Catarina (Kuhnen et al., 2012; Cardoso, 2018; Costa et al., 2019, 2022; Kersul et al., 2020; Boullosa et al., 2020; Lucio et al., 2021). These studies revealed the importance of phylogenetic, biotic and abiotic factors in the structuring of parasite helminth communities. However, there is still a large gap in knowledge concerning the diversity of this group of parasites in rodents (Cardoso et al., 2020; Costa et al., 2022), especially in areas of Araucaria forests in southern Brazil (Benatti et al., 2021).

## Objectives

The objective of this study was to describe the helminth communities of rodents in the municipalities of Irati, Balsa Nova and Guarapuava, state of Paraná, Brazil, and to compare helminth population parameters in relation to host sex, age and body size.

## Materials and Methods

### Study area

**Figure 1:**
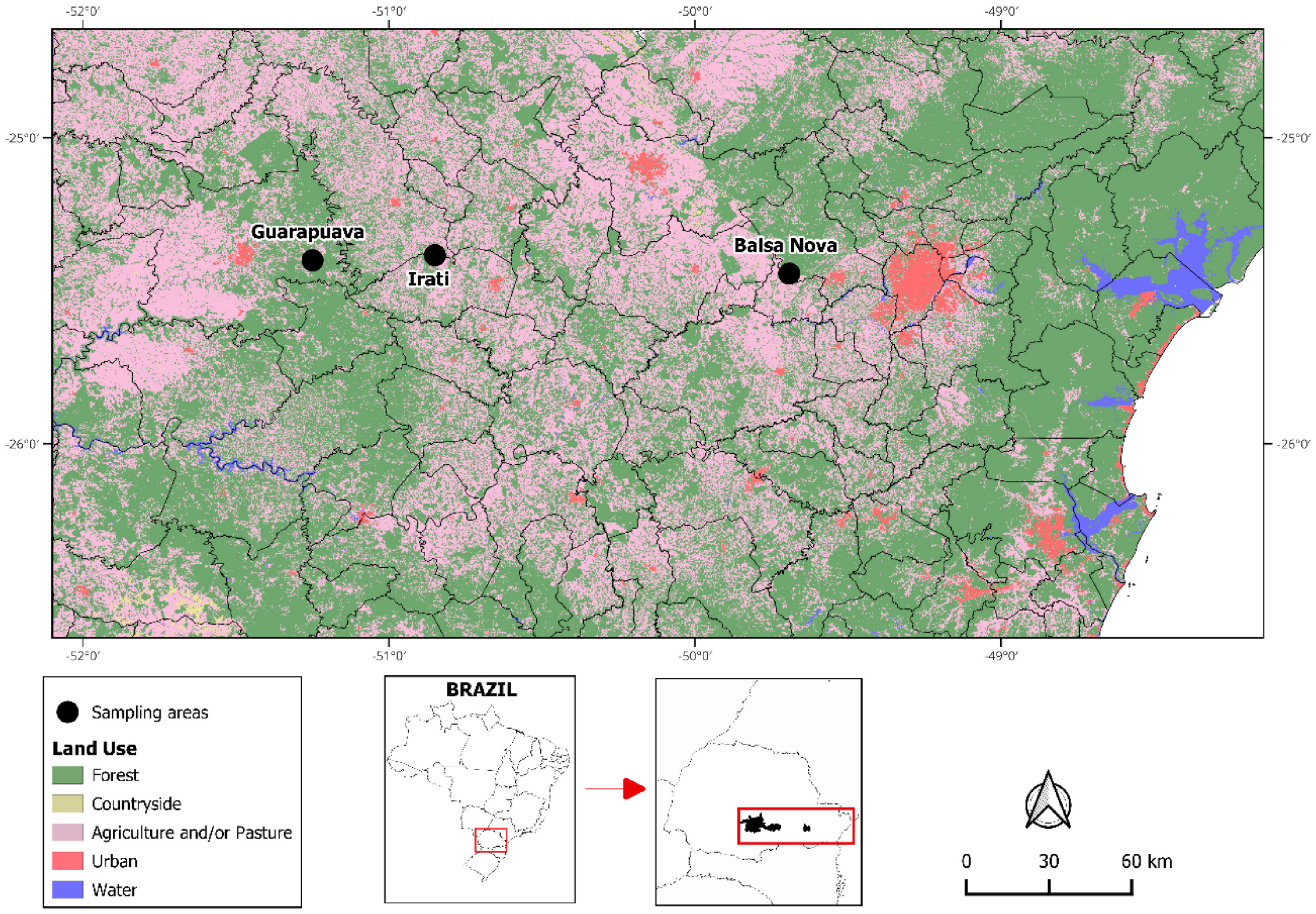
Locations of the municipalities of Guarapuava, Irati and Balsa Nova in the state of Paraná, Brazil, where rodents were collected, including the land use of the region.

The present study was conducted in the municipalities of Irati, Balsa Nova and Guarapuava, state of Paraná, Brazil (Figure 1) from late March to early July 2023. The three localities are within the Atlantic Forest biome in the domain of the mixed ombrophylous forest (Araucaria Forest). All the study areas shared similar characteristics, including edges and interiors of Araucaria forests (*Araucaria angustifolia*), a vegetation known as Mixed Ombrophilous Forest, adjacent to peridomicile areas, grasslands and agricultural fields of sugarcane, wheat and soybean.

### Hosts

The hosts used in this study were rodents of the species *Akodon montensis, Akodon paranaensis, Euryoryzomys russatus, Mus musculus, Oligoryzomys nigripes, Rattus rattus,* and *Thaptomys nigrita*. All the hosts inhabit forest formations of the Atlantic Forest. The rodent *Akodon montensis* Thomas, 1913 is terrestrial, has an insectivorous/omnivorous diet, and is distributed along the southern coast of Brazil (Bonvicino et al., 2008; Paglia et al, 2012).

*Akodon paranaensis* Christoff, Fagundes, Sbalqueiro, Mattevi & Yonenaga-Yassuda, 2000 is found in southern and southeastern Brazil and extends into Argentina and Paraguay (Patton et al., 2015). It has an insectivorous/omnivorous diet (Paglia et al., 2012). *Euryoryzomys russatus* (Wagner, 1848) has a terrestrial habit and a frugivorous/granivorous diet (Bonvicino et al., 2008; Paglia et al, 2012).

*Mus musculus* (Linnaeus, 1758) occurs near urban dwellings and is present in all states. It can occur in two forms: as a commensal, in which it depends on humans for food and shelter, and lives far from human dwellings in the wildlife. (Bonvicino et al., 2008; Gray & Hurst, 1998; Berry & Scriven, 2005). *Oligoryzomys nigripes* (Olfers,1818) is terrestrial and has a frugivorous/granivorous diet. (Bonvicino et al., 2008; Paglia et al, 2012).

*Rattus rattus* (Linnaeus, 1758) is a terrestrial, commensal and generalist rodent. It has the ability to adapt easily to a wide range of environments and resources (Bonvicino et al., 2008; Aplin et al., 2003). *Thaptomys nigrita* (Lichtenstein, 1829) is terrestrial and has an insectivorous/omnivorous diet. (Bonvicino et al., 2008; Paglia et al, 2012).

### Collection of rodents and helminths

Linear transects of 20 points were established at each locality for rodent capture, resulting in a total capture effort of 600 traps-nights in each municipality, such that the trapping effort was the same in all localities. Each point contained a Sherman live-trap (7.62 cm × 9.53 cm × 30.48 cm). All the samplings were carried out in the autumn of 2023, during five consecutive days. Each locality was sampled only once. The traps were baited with banana, peanut butter, bacon and oats. Rodents were captured under authorization of the Brazilian Government’s Chico Mendes Institute for Biodiversity and Conservation (ICMBIO, Licence 13373). All procedures followed the guidelines for the capture, handling, and care of animals of the Ethical Committee on Animal Use of the Oswaldo Cruz Foundation (CEUA, Licence L-036/2018-A1) and followed biosafety protocols for the capture and handling of wild animals (Lemos & D'Andrea, 2014). The collected animals were submitted to taxidermy, and their skeletons were prepared and deposited as voucher material in the Integrated Collection of Wild Mammal Reservoirs (COLMASTO) of the Oswaldo Cruz Institute. The rodents were identified using an integrated approach that included external morphology, cytogenetic analysis (karyotyping) and molecular sequencing.

The organs (stomach, small intestine and large intestine) were placed in individual Petri dishes and washed in saline solution (0.85% NaCl). The recovered helminths were placed in Petri dishes with saline solution (0.85% NaCl). The specimens were fixed and kept in ethanol (Amato et al., 1991) and stored in glass tubes labelled with a standard label that were stored in the helminthological collection of the Laboratory of Biology and Parasitology of Wild Mammal Reservoirs at the Oswaldo Cruz Institute.

### Identification of helminths

The helminths were identified using a light microscope (Axio Scope. A1 – Zeiss coupled to an AxioCam MRc digital camera) for photomicrography. The nematodes were diaphanized with lactophenol and placed between a slide and coverslip for identification by light microscopy. Cestodes were stained with Langeron Carmine, differentiated with 0.5% hydrochloric alcohol, dehydrated in an increasing alcoholic series, diaphanized in methyl salicylate and permanently mounted in Canada Balsam, with permanent preparation (Amato et al., 1991). Helminths were identified at the species level, when possible, counted and separated according to sex (except in hermaphrodites). The specific morphological aspects used in the identification of the specimens followed Travassos (1937), Yamaguti (1961), Vicente et al. (1997), Khalil et al. (1994) and others, in addition to the articles describing related species. Voucher specimens were deposited at the Helminthological Collection of the Oswaldo Cruz Institute (Appendix 1).

### Data analysis

The mean abundances and mean intensities and their standard deviations and the prevalence and confidence intervals of each helminth species were calculated according to Bush et al. (1997) for each host species and locality. Richness was considered the mean number of helminth species recovered from each host species.

The most abundant rodent species were analysed separately to evaluate the influence of host sex, age, body size and locality on the total richness and abundance of helminths per infracommunity (helminth community of each individual host). Host age was divided into young and adult according to the body size, body weight and reproductive condition of the animals. We considered as young the animals whose body size and body weight were below the minimum values observed for the reproducing animals**.** Sex, age and locality were tested using the t test or Mann‒Whitney test according to the data distribution. The data distribution was tested using the Shapiro‒Wilk test. The influence of host body size on the total abundance of helminths was tested using linear regression. The analyses were performed using the software Past version 4.12 (Hammer et al., 2001). In all analyses, a significance level of 5% was considered.

## Results

Among the 143 hosts analysed, 66 were positive for the presence of helminths. Thirty-three hosts were *Akodon montensis* Thomas, 1913, five were *Akodon paranaensis* Christoff, Fagundes, Sbalqueiro, Mattevi and Yonenaga-Yassuda, 2000, four were *Euryoryzomys russatus* (Wagner, 1848), 11 were *Oligoryzomys nigripes* (Olfers, 1818), two were *Thaptomys nigrita* (Lichtenstein, 1829) (Cricetidae, Sigmodontinae), ten were *Mus musculus* (Linnaeus, 1758) and one was *Rattus rattus* (Linnaeus, 1758) (Muridae, Murinae). The species *Oxymycterus quaestor* (Thomas, 1903) was also collected*;* however, no helminths were found in this species (Table 1).

**Table 1:** Number of hosts analysed and positive, and prevalence for the presence of helminths from Irati, Balsa Nova and Guarapuava, state of Paraná, Brazil, in 2023.

					LOCALITY						
**Host**	**Guarapuava**					**Balsa Nova**			**Irati**		
	**Analysed**	**Positive**		**Prevalence**		**Analysed**	**Positive**	**Prevalence**	**Analysed**	**Positive**	**Prevalence**
*Akodon montensis*	17	8		47.05%		12	6	50%	28	19	67.85%
*Akodon paranaensis*	8	5		62.50%		7	0	0%	1	0	0%
*Euryoryzomys russatus*	-	-		-		6	4	66.66%	-	-	-
*Mus musculus*	42	10		23.80%		-	-	-	-	-	-
*Oligoryzomys nigripes*	1	1		100%		12	10	83.3%	3	0	0%
*Oxymycterus quaestor*	2	0		0%		1	0	0%	-	-	-
*Rattus rattus*	1	1		100%		-	-	-	-	-	-
*Thaptomys nigrita*	1	1		100%		1	1	100%	-	-	-
**Total**	**72**	**26**		**36.11%**		**39**	**21**	**53.84%**	**32**	**19**	**59.37%**

**Figure 2:**
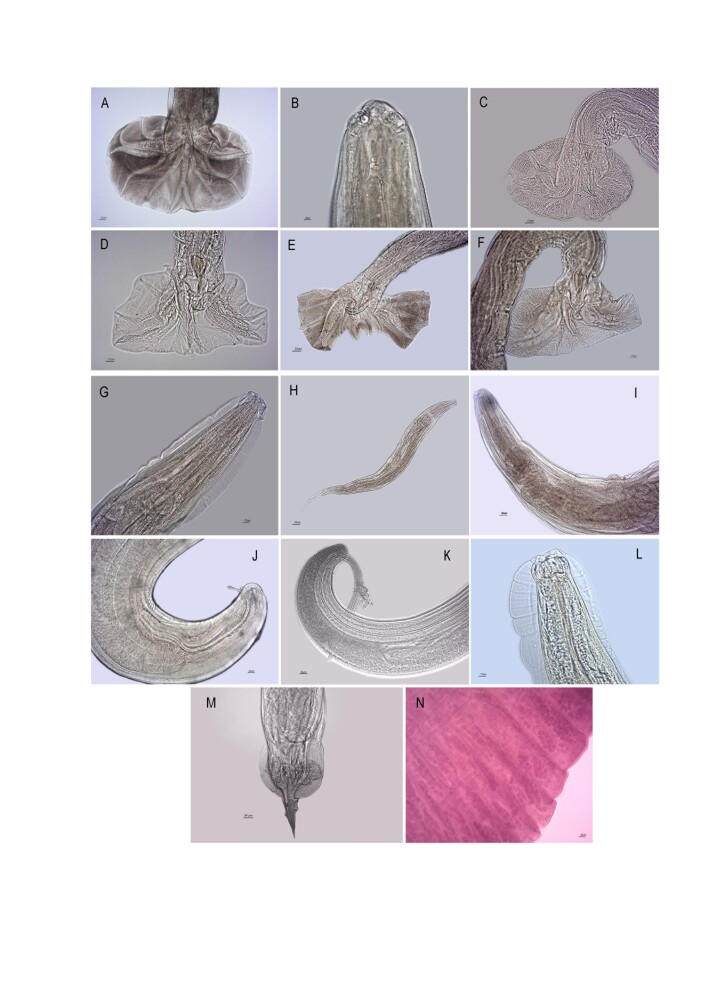
Morphological aspects of the helminths found in rodents captured in three areas of the Atlantic rainforest in the Paraná state, Brazil. (A) *Guerrerostrongylus zeta*; (B) *Protospirura numidica criceticola*; (C) *Stilestrongylus aculeata*; (D) *Stilestrongylus eta*; (E) *Stilestrongylus lanfrediae*; (F) *Stilestrongylus rolandoi*; (G) *Syphacia carlitosi*; (H) *Syphacia kinsellai*; (I) *Syphacia evaginata*; (J) *Trichuris muris*; (K) *Trichuris navonae*; (L) *Pterygodermatites* sp.; (M) *Heterakis spumosa*; (N) Cestoda sp. 2.

A total of 937 helminth specimens were found. From the phylum Nematoda, the following species were identified: *Guerrerostrongylus zeta* (50), *Protospirura numidica criceticola* (4), *Stilestrongylus aculeata* (54), *Stilestrongylus eta* (285), *Stilestrongylus lanfrediae* (49), *Stilestrongylus rolandoi* (82)*, Stilestrongylus freitasi* (14)*, Syphacia carlitosi* (174), *Syphacia kinsellai* (9), *Syphacia evaginata* (21), *Trichuris muris* (5)*, Trichuris navonae* (5) and *Heterakis spumosa* (92). The unidentified species *Pterygodermatites* sp. (2), *Stilestrongylus* sp. 1 (4), *Stilestrongylus* sp. 2 (5), *Stilestrongylus* sp. 3 (1), *Syphacia* sp. 1 (23), *Syphacia* sp. 2 (1), *Syphacia* sp. 3 (4), *Syphacia* sp. 4 (26), *Syphacia* sp. 5 (12), *Syphacia* sp. 6 (2), *Trichuris* sp. 1 (1), *Trichuris* sp. 2 (2), and *Trichuris* sp. 3 (1) of the phylum Nematoda were also recorded. These morphotypes could not be identified at the species level because there were no male individuals in the samples and/or the material was not in good condition. In addition, from the phylum Platyhelminthes, three unidentified species of cestodes were found, Cestoda (1), Cestoda sp. 1 (3) and Cestoda sp. 2 (5), which could not be identified at the species level because of the absence of the scolex (Figure 2).

For the rodent *A. montensis, S. aculeata* had the highest abundance and intensity in the locality of Irati (Table 2). This helminth species occurred only in this locality. The highest prevalence of helminths in *A. montensis* from Irati was observed for Cestoda sp. 2, which also occurred in Balsa nova. However, the most prevalent helminths for *A. montensis* in Balsa Nova were *S. carlitosi* and *H. spumosa*, the latter of which also presented greater abundance and intensity. In Guarapuava, *S. carlitosi* was the most abundant species, and *G. zeta* was the most prevalent in *A. montensis* (Table 2)*.* The helminth species richness ranged from 0 to 2, with a mean of 0.62 ± 0.74.

**Table 2:** Mean abundance and intensity of helminths ± standard deviation, minimum and maximum values of abundance and intensity, and helminth prevalence (95% confidence interval) in the rodent *Akodon montensis* in Irati, Balsa Nova and Guarapuava, state of Paraná, Brazil.

Species	*Guerrerostrongylus zeta*		*Heterakis spumosa*		*Protospirura numidica criceticola*	*Stilestrongylus aculeata*		*Stilestrongylus eta*		*Stilestrongylus lanfrediae*		*Stilestrongylus* sp. 1		*Stilestrongylus* sp. 2		*Syphacia carlitosi*
**IRATI**						**N = 54**		**N = 28**		**N = 7**		**N = 4**		**N = 5**		**N = 22**
Mean Abundance	**-**		-		-	1.93 ± 6.46 (0-26)		1.00 ± 3.91 (0-20)		0.25 ± 0.93 (0-4)		0.14 ± 0.76 (0-4)		0.18 ± 0.94 (0-5)		0.79 ± 3.59 (0-19)
Mean Intensity	**-**		-		-	18.00 ± 11.36 (1-3)		9.33 ± 9.45 (1-3)		3.50 ± 0.71 (1-2)		4.00 ± 0.00 (1-1)		5.00 ± 0.00 (1-1)		7.33 ± 10.12 (1-3)
Prevalence	-		-		-	10.71 (2.27 – 28.23)		10.71 (2.27 – 28.23)		7.14 (0.88 – 23.50)		3.57 (0.09 – 18.35)		3.57 (0.09 – 18.35)		10.71 (2.27 – 28.23)
**BALSA NOVA**			**N = 85**													**N = 8**
Mean Abundance	-		6.00 ± 15.58 (0-52)		-	-		-		-		-		-		0.62 ± 1.94 (0-1)
Mean Intensity	-		39.00 ± 18.38 (1-2)		-	-		-		-		-		-		4.00 ± 0.00 (1-1)
Prevalence	-		15.38 (1.92 – 45.45)		-	-		-		-		-		-		15.38 (1.92 – 45.45)
**GUARAPUAVA**	**N = 26**				**N = 3**											**N = 37**
Mean Abundance	1.53 ± 4.35 (0-17)		**-**		0.18 ± 0.53 (0-2)	-		-		-		-		-		2.18 ± 8.22 (0-34)
Mean Intensity	8.67 ± 7.64 (1-3)		**-**		1.50 ± 0.71 (1-2)	-		-		-		-		-		12.33 ± 18.77 (1-3)
Prevalence	17.65 (3.8 – 43.43)		**-**		11.76 (1.46 – 36.44)	-		-		-		-		-		17.65 (3.8 – 43.43)
Species		*Syphacia kinsellai*		*Syphacia* sp. 2			*Trichuris navonae*		*Trichuris* sp. 1		*Trichuris* sp. 2		Cestoda sp. 1		Cestoda sp. 2	
**IRATI**		**N = 4**							**N = 1**		**N = 2**				**N = 4**	
Mean Abundance		0.14 ± 0.76 (0-4)		-			-		0.04 ± 0.19 (0-1)		0.07± 0.38 (0-2)		-		0.14 ± 0.36 (0-1)	
Mean Intensity		4.00 ± 0.00 (1-1)		-			-		1.00 ± 0.00 (1-1)		2.00 ± 0.00 (1-1)		-		1.00 ± 0.00 (1-4)	
Prevalence		3.57 (0.09 – 18.35)		-			-		3.57 (0.09 – 18.35)		3.57 (0.09 – 18.35)		**-**		14.29 (4.03 – 32.67)	
**BALSA NOVA**							**N = 2**								**N = 1**	
Mean Abundance		-		-			0.15 ± 0.55 (0-2)		-		-		-		0.08 ± 0.28 (0-1)	
Mean Intensity		-		-			2.00 ± 0.00 (1-1)		-		-		-		1.00 ± 0.00 (1-1)	
Prevalence		-		-			7.69 (0.19 – 36.03)		-		-		-		7.69 (0.19 – 36.03)	
**GUARAPUAVA**				**N = 1**			**N = 3**						**N = 1**			
Mean Abundance		-		0.06 ± 0.24 (0-1)			0.18 ± 0.73 (0-3)		-		-		0.06 ± 0.24 (0-1)		-	
Mean Intensity		-		1.00 ± 0.00 (1-1)			3.00 ± 0.00 (1-1)		-		-		1.00 ± 0.00 (1-1)		-	
Prevalence		-		5.88 (0.15 – 28.69)			5.88 (0.15 – 28.69)		-		-		5.88 (0.15 – 28.69)		-	

The dash (-) indicates absence. N (total number of individuals).

For the host *A. paranaensis,* only *H. spumosa* and *S. carlitosi* were recorded in Balsa Nova. In Guarapuava, *Syphacia* sp. 4 had the greatest abundance and intensity and Cestoda sp. 1 had the highest prevalence (Table 3). The species richness ranged from 0 to 3, with a mean of 0.67 ± 0.90.

**Table 3:** Mean abundance and intensity of helminths ± standard deviation, minimum and maximum values of abundance and intensity, and helminth prevalence (95% confidence interval) in the rodent *Akodon paranaensis* in Balsa Nova and Guarapuava, state of Paraná, Brazil.

Species	*Guerrerostrongylus zeta*	*Heterakis spumosa*	*Protospirura numidica criceticola*	*Stilestrongylus lanfrediae*	*Syphacia carlitosi*	*Syphacia* sp. 4	Cestoda sp. 1
**BALSA NOVA**		**N = 7**			**N = 7**		
Mean Abundance	-	1.00 ± 2.65 (0-7)	-	-	1.00 ± 2.65 (0-7)	**-**	-
Mean Intensity	-	7.00 ± 00.00 (1-1)	-	-	7.00 ± 0.00 (1-1)	**-**	-
Prevalence	-	14.29 (0.36 – 57.87)	-	-	14.29 (0.36 – 57.87)	**-**	-
**GUARAPUAVA**	**N = 9**		**N = 1**	**N = 8**	**N = 1**	**N = 26**	**N = 2**
Mean Abundance	1.13 ± 3.18 (0-9)	-	0.13 ± 0.35 (0-1)	1.00 ± 2.83 (0-8)	0.13 ± 0.35 (0-1)	3.13 ± 8.84 (0-25)	0.25 ± 0.46 (0-1)
Mean Intensity	9.00 ± 0.00 (1-1)	-	1.00 ± 0.00 (1-1)	8.00 ± 0.00 (1-1)	1.00 ± 0.00 (1-1)	25.00 ± 0.00 (1-1)	0.67 ± 0.00 (1-3)
Prevalence	12.5 (0.32 – 52.65)	-	12.5 (0.32 – 52.65)	12.5 (0.32 – 52.65)	12.5 (0.32 – 52.65)	12.5 (0.32 – 52.65)	37.50 (8.52 – 75.51)

The dash (-) indicates absence. N (total number of individuals).

The host *E. russatus* occurred only in Balsa Nova, and three helminth species were found, with the highest abundances for *S. eta* and *S. rolandoi,* the latter occurring only in this host. *S.eta* and Cestoda were the most prevalent in *E. russatus* (Table 4). The species richness ranged from 0 to 2, with a mean of 0.83 ± 0.75.

**Table 4:** Mean abundance and intensity of helminths ± standard deviation, minimum and maximum values of abundance and intensity, and helminth prevalence (95% confidence interval) in the rodent *Euryoryzomys russatus* Balsa Nova, state of Paraná, Brazil.

Species	*Stilestrongylus eta*	*Stilestrongylus rolandoi*	Cestoda
**BALSA NOVA**	**N = 82**	**N = 82**	**N = 1**
**Mean Abundance**	13.67 ± 33.48 (0-82)	13.67 ± 16.74 (0-41)	0.17 ± 0.41 (0-1)
**Mean Intensity**	82.00 ± 0.00 (1-1)	27.33 ± 11.85 (1-3)	1.00 ± 0.00 (1-1)
**Prevalence**	16.67 (0.42 – 64.12)	50 (11.81 – 88.19)	16.67 (0.42 – 64.12)

The dash (-) indicates absence. N (total number of individuals).

The host *M. musculus* occurred only in Guarapuava. The helminths *S. eta*, *S. freitasi*, *S. evaginata* and *Syphacia* sp. 5 were found in this host. *Stilestrongylus eta* had the highest abundance, intensity and prevalence (Table 5). The species richness ranged from 0 to 2, with a mean of 0.33 ± 0.65.

**Table 5:** Mean abundance and intensity of helminths ± standard deviation, minimum and maximum values of abundance and intensity. and helminth prevalence (95% confidence interval) in the rodent *Mus musculus* in Guarapuava. state of Paraná, Brazil.

Species	*Stilestrongylus eta*	*Stilestrongylus freitasi*	*Syphacia evaginata*	*Syphacia* sp. 5
**GUARAPUAVA**	**N = 175**	**N = 14**	**N = 12**	**N = 12**
Mean Abundance	4.17 ± 18.74 (0-119)	0.33 ± 2.16 (0-14)	0.29 ± 1.20 (0-7)	0.29 ± 1.29 (0-8)
Mean Intensity	25.00 ± 42.32 (1-7)	14.00 ± 2.00 (1-1)	4.00 ± 2.65 (1-3)	4.00 ± 3.46 (1-3)
Prevalence	16.67 (6.97 – 31.36)	2.38 (0.06 – 12.57)	7.14 (1.05 – 19.48)	7.14 (1.05 – 19.48)

The dash (-) indicates absence. N (total number of individuals).

Only one individual of *R. rattus* was collected from Guarapuava. This animal was infected with *T. muris* (5 specimens) and *Syphacia* sp. 6 (2 specimens).

In *O. nigripes,* the helminth *S. carlitosi* had the greatest abundance and intensity in Balsa Nova, and *G. zeta, S. lanfrediae* and *Syphacia* sp. 1 had the highest prevalence (Table 6). In Guarapuava, only two helminth species were found in *O. nigripes,* with *G. zeta* having the highest abundance, intensity and prevalence (Table 6). The species richness ranged from 0 to 3, with a mean of 1 ± 0.89.

**Table 6:** Mean abundance and intensity of helminths ± standard deviation, minimum and maximum values of abundance and intensity, and helminth prevalence (95% confidence interval) in the rodent *Oligoryzomys nigripes* in Balsa Nova and Guarapuava, state of Paraná, Brazil.

Species	*Guerrerostrongylus zeta*	*Pterygodermatites* sp.	*Stilestrongylus lanfrediae*	*Stilestrongylus* sp. 3	*Syphacia carlitosi*	Syphacia *evaginata*	*Syphacia* sp. 1	*Syphacia* sp. 3
**BALSA NOVA**	**N = 7**	**N = 2**	**N = 34**	**N = 1**	**N = 85**	**N = 9**	**N = 23**	
Mean Abundance	0.58 ± 1.16 (0-3)	0.17 ± 0.58 (0-2)	2.83 ± 6.78 (0-23)	0.08 ± 0.29 (0-1)	7.08 ± 24.54 (0-85)	0.75 ± 0.00 (0-9)	1.92 ± 4.08 (0-13)	-
Mean Intensity	2.33 ± 1.15 (1-3)	2.00 ± 0.00 (1-1)	11.33 ± 10.41 (1-3)	1.00 ± 0.00 (1-1)	85.00 ± 0.00 (1-1)	9.00 ± 0.00 (1-1)	7.67 ± 5.03 (1-3)	-
Prevalence	25 (5.49 – 57.19)	8.33 (0.21 – 38.48)	25 (5.49 – 57.19)	8.33 (0.21 – 38.48)	8.33 (0.21 – 38.48)	8.33 (0.21 – 38.48)	25 (5.49 – 57.19)	-
**GUARAPUAVA**	**N = 8**							**N = 4**
Mean Abundance	8.00 ± 0.00 (0-8)	-	**-**	-	-	-	-	4.00 ± 0.00 (0-4)
Mean Intensity	8,00 ± 0,00 (1-1)	-	**-**	-	-	-	-	4,00 ± 0,00 (1-1)
Prevalence	100 (2,5 – 100)	-	**-**	-	-	-	-	100 (2,5 – 100)

The dash (-) indicates absence. N (total number of individuals).

Only two individuals of the rodent *T. nigrita* were collected*,* one from Balsa Nova and one from Guarapuava. The species *S. carlitosi* was found in both localities, and *Trichuris* sp. 3 was found only in Balsa Nova. The abundance of *S. carlitosi* was 12 in Guarapuava and seven in Balsa Nova. A single specimen of *Trichuris* sp. 3 was found in this host.

Some helminth species were associated with more than one host species. *Guerrerostrongylus zeta* was found in *O. nigripes* at the locality of Balsa Nova and Guarapuava and in *A. montensis* and *A. paranaensis* at the latter locality. *Heterakis spumosa* was found in *A. montensis* and *A. paranaensis* at Balsa Nova. *Protospiura numidica criceticola* was found in *A. montensis* and *A. paranaensis* at Guarapuava. *Stilestrongylus eta* was present in *A. montensis* (Irati), *M. musculus* (Guarapuava) and *E. russatus* (Balsa Nova). *Stilestrongylus lanfrediae* was present in *A. montensis* (Irati), *O. nigripes* (Balsa Nova) and *A. paranaensis* (Guarapuava). *Syphacia carlitosi* occurred in *A. montensis* (Irati, Balsa Nova and Guarapuava), *A. paranaensis* (Balsa Nova and Guarapuava), *O. nigripes* (Balsa Nova) and *T. nigrita* (Balsa Nova and Guarapuava). *Syphacia evaginata* occurred in *O. nigripes* (Balsa Nova) and *M. musculus* (Guarapuava). *Syphacia kinsellai* occurred in *A. montensis* (Irati) and *O. nigripes* (Balsa Nova).

The analyses to evaluate the influence of intrinsic factors (host age and sex) and locality on total species richness and total abundance of the helminths were performed only for the most abundant host species, separately, which were *A. montensis*, *A. paranaensis*, *O. nigripes* and *M. musculus*. No significant differences were found for the analysed variables (sex, age, or locality) for *O. nigripes*, *A. paranaensis* or *M. musculus* (Table 7). Only for *A. montensis,* a significant difference was found in relation to host age considering total helminth abundance, with greater abundance observed in young hosts (Table 7). No relationship was found between host body size and total helminth abundance for any of the host species analysed (*O. nigripes*: β = -49.17; r = 0.669; p = 0.232; *A. montensis*: β = -0.039; r= -0.042; p=0.75; *A. paranaenis*: β = -0.078; r= -0.0152; p = 0.570; *M. musculus*: β = 0.429; r = 0.158; p = 0.316).

**Table 7:** Results of the t and Mann‒Whitney tests of the total helminth abundance in relation to host sex and age and locality in Balsa Nova (B), Guarapuava (G) and Irati (I), state of Paraná, Brazil.

**Hosts**	**Sex**		**Age**		**Locality**	
***Oligoryzomys nigripes***	**M = 6**	**F = 10**	**Y= 2**	**A= 14**	**BN = 10 G = 1 I = 3**	
Abundance	z = 0.601	p = 0.544	-	-	-	-
Species Richness	teste t = 1.169	p = 0.262	-	-	-	-
***Akodon montensis***	**M = 36**	**F = 22**	**Y = 20**	**A = 38**	**BN = 10 G = 14 I = 14**	
Abundance	z = 1.290	p = 0.197	z = 2.064	p = 0.039^*^	h = 0.591	p =0.705
Species Richness	z =1.400	p = 0.161	z = 1.405	p =0.160	h = 0.984	p = 0.546
***Akodon paranaensis***	**M = 4**	**F = 11**	**Y = 8**	**A = 7**	**BN = 7 G = 8 I = 0**	
Abundance	z = 0.099	p = 0.321	z = 0.126	p = 0.900	p = 0.224	p = 0.224
Species Richness	z = 0.579	p = 0.562	z = 0.129	p = 0.897	z = 0.984	p = 0.325
***Mus musculus***	**M = 22**	**F = 19**	**Y = 19**	**A = 23**	**BN = 0 G = 23 I = 0**	
Abundance	z = 0.132	p = 0.895	z = 0.354	p = 0.723	-	-
Species Richness	z = 0.099	p = 0.921	z = 0.354	p = 0.723	-	-

* Significant difference.

The dash (-) indicates absence. M – males, F – females, Y – young, A – adult, BN – Balsa Nova, G – Guarapuava, I – Irati.

## Discussion

Considering the species composition of the helminth communities of the rodents analysed in the three municipalities of the state of Paraná, Brazil, the phylum Nematoda was the most abundant, represented by six genera. Nematodes are an evolutionarily successful group representing the most abundant metazoans in the world (Bongers & Ferris, 1999; Blaxter & Koutsovoulos, 2015). In other studies of the helminth fauna of sigmodontine rodents, nematodes were also the most abundant group (Lucio, 2019; Cardoso, 2018; Simões, 2009; Boullosa et al., 2020). The absence of trematodes may be because none of these rodents is semi-aquatic.

In the present study, the helminth fauna of *A. montensis* comprised 16species. A study conducted in two localities in the western region of the state of Paraná (Corbélia and Cascavel) *A. montensis* presented ten helminth species (Benatti et al., 2021), including *P. n. criceticola*, *S. aculeata*, *S. eta*, and *T. navonae*, which were also found in the present study. A study conducted in Serra dos Órgãos National Park, state of Rio de Janeiro, reported seven species for this host, presenting the same helminths in common with those reported by Benatti et al. (2021), in addition to *S. lanfrediae,* present in this study. *Stilestrongylus lanfrediae* was originally described in *O. nigripes* in Serra dos Órgãos National Park, state of Rio de Janeiro (Souza et al., 2009). The helminth species with the highest intensity and abundance were those of the genus *Stilestrongylus* (Cardoso et al., 2019), as was observed in the locality of Irati, with the highest intensity and abundance of *S. aculeata*. In a study conducted in Santa Catarina, six species of helminths were found in *A. montensis*, including *G. zeta* and *T. navonae* (Boullosa et al., 2020), which were also found in the present study. Lucio et al. (2025) reported nine helminth species in *A. montensis* in preserved and altered areas of the Atlantic Forest in the state of São Paulo. They reported all the species cited in the above studies, in addition to *S. carlitosi* (Lucio et al., 2025)*.*

*Heterakis spumosa* is commonly found in birds that feed on the ground and rarely in mammals (Ribas et al., 2013). When found in rodents, this helminth is more common in cosmopolitan species, such as *R. rattus*, *Rattus norvegicus*, and *M. musculus,* and most likely originated in Asia (Poonlaphdecha et al., 2024). Thus, the results of this study indicate that *A. montensis* and *A. paranaensis* are new hosts for *H. spumosa*, expanding their lists of hosts. In contrast to expectations, this helminth species was not found in *M. musculus* or *R. rattus* in this study.

Studies regarding the helminth fauna of *A. paranaensis* are scarce, with only one unpublished study conducted in areas of high-elevation grasslands in Itatiaia National Park, state of Rio de Janeiro, where six nematode species and one cestode were identified (SIMÕES, personal communication). In the present study, seven helminth species were found in this host.

The rodent *O. nigripes* had four identified helminth species and four unidentified species. *Guerrerostrongylus zetta* and *S. lanfrediae* were also reported by Boullosa et al. (2020) in the state of Santa Catarina, who found only these species, and by Cardoso et al. (2020) in the state of Rio de Janeiro, who reported four helminth species in this host. In the study of Boullosa et al. (2020), the abundance, intensity and prevalence of the species *G. zeta* were the highest, a pattern similar to that observed in the present study in the locality of Guarapuava. *Syphacia* sp., which was found in the present study, was also found by Kersul et al. (2020) in the state of Bahia, who recorded three helminth species in *O. nigripes*, and by Graciano (2022) in the state of Minas Gerais, who reported four species in this host. The present study is the first report of *S. carlitosi* and *S. evaginata* and the genus *Pterygodermatites* in *O. nigripes*.

The rodent *M. musculus* had three identified helminth species and one unidentified in the locality of Guarapuava in the present study. *Stilestrongylus freitasi* was also found in this host in a study conducted at the Poço das Antas Biological Reserve in the state of Rio de Janeiro (Lucio, 2019), which recorded three helminth species. *Syphacia evaginata* was also reported in this host in other localities of the state of Paraná (Corbélia and Cascavel), where the authors reported four helminth species (Benatti et al., 2021). The present study is the first report of *S. eta* in *M. musculus*.

*Euryoryzomys russatus* had three helminth species in Balsa Nova, with the highest abundance of *S. rolandoi*. This high prevalence corroborates the findings of Lucio et al. (2025), who reported two species in this host in the state of São Paulo, and Boullosa et al. (2020), who reported three species in the state of Santa Catarina. The present study is the first report of *S. eta* in *E. russatus.* The cestode species previously reported for *E. russatus* was *Raillietina guaricanae* (Boullosa et al., 2020); however, it was not possible to identify the cestode species in the present study.

This study is the first report of *S. carlitosi* and the genus *Trichuris* in the host *T. nigrita*. Compared with those of previous studies, Benatti et al. (2021) reported two species in other localities of the state of Paraná (Corbélia and Cascavel), *Syphacya criceti* and *Stilestrongylus* sp.. In a study conducted in the state of Rio de Janeiro, Cardoso et al. (2020) reported *P. n. criceticola, Stilestrongylus* sp. and *Pterygodermatites* sp.. In localities of the state of São Paulo, Lucio (2025) reported two species in this host, *P. n. criceticola* and *Syphacia hugoti*.

*Trichuris muris*, which was found in *R. rattus,* was previously reported in this host by Muñoz et al. (2018) in Argentina. Panti-May et al. (2019) and Gentile et al. (2022) reported *Syphacia muris* in this host.

Although most parasites are host specialists (Combes, 2001), parasite sharing can be common among hosts, especially congeners or phylogenetically related species that present similar functional traits (Cardoso et al., 2021), as the same factors may drive host-parasite interactions. Thus, the helminth sharing observed among some hosts in this study may be attributed to 1) their close taxonomic distance, as most of the helminth shares were among sigmodontine rodents, especially between *A. montensis* and *A. paranaensis*, and 2) the similar characteristics of the habitat among localities providing favorable soil conditions for the helminth cycles.

The analyses of the influence of intrinsic (sex, age and body size) and extrinsic (locality) factors on total helminth species richness and abundance did not reveal significant differences for most of the species analysed. Only in *A. montensis,* helminth abundance was significantly different between young and adult hosts, with young individuals presenting a greater total abundance of helminths than adults did. It is expected that adult hosts have a greater parasite load, both because of the longer exposure time and consequent accumulation of specimens throughout life and because of their larger body size (Behnke et al., 1999; Cardoso et al., 2019; Cirino et al., 2020). However, these patterns were not observed in the present study. The higher helminth abundance observed in young individuals of *A. montensis* may be related to the small sample size and the fact that most of the animals captured were adults, which are limiting factors of the present study.

No differences in helminth species richness or abundance were observed among localities, possibly because they have very similar environmental characteristics. Irati, Balsa Nova and Guarapuava are part of the Atlantic Forest biome and have similar climates, phytophysiognomies and types of land use. In addition, these areas have a certain degree of anthropization and are close to human habitations, although the percentage of preserved vegetation cover differs among the localities (Projeto MapBiomas, 2025).

This study provides a novel contribution to the knowledge of the diversity of helminths in rodents. This study provides new information about the occurrence of some helminth species, increases the host species list, geographic distribution, and parasitological parameters of some helminth species, contributes to the mapping of Brazilian parasite fauna and highlights the importance of regional studies for understanding the ecology of parasites.

## Data Availability

The raw data supporting the results of this study are available upon request from the authors.

## Appendix 1 Voucher specimens deposited in the Helminthological Collection of the Oswaldo Cruz Institute (CHIOC).

**Table:**

**Species**	**CHIOC numbers**	**Locality**
***Akodon montensis***		
*Heterakis spumosa*	39828	Balsa Nova - PR
*Syphacia carlitosi*	39829	Balsa Nova - PR
*Trichuris navonae*	39826	Balsa Nova - PR
Cestoda sp. 2	39827	Balsa Nova - PR
*Protospirura numidica criceticola*	39831	Guarapuava - PR
*Syphacia carlitosi*	39833	Guarapuava - PR
*Guerrerostrongylus zeta*	39834	Guarapuava - PR
*Trichuris navonae*	39832	Guarapuava - PR
*Syphacia* sp. 2	39835	Guarapuava - PR
Cestoda sp. 1	39830	Guarapuava - PR
*Syphacia carlitosi*	39818	Irati - PR
*Syphacia kinsellai*	39824	Irati - PR
*Stilestrongylus aculeata*	39822	Irati – PR
*Stilestrongylus eta*	39821	Irati – PR
*Stilestrongylus lanfrediae*	39823	Irati – PR
*Stilestrongilus* sp.1	39819	Irati – PR
*Stilestrongylus* sp.2	39820	Irati – PR
Cestoda sp. 2	39825	Irati – PR
***Akodon paranaensis***		
*Heterakis spumosa*	39841	Balsa Nova - PR
*Syphacia carlitosi*	39842	Balsa Nova - PR
*Syphacia carlitosi*	39837	Guarapuava - PR
*Guerrerostrongylus zeta*	39839	Guarapuava - PR
*Syphacia* sp. 4	39836	Guarapuava - PR
*Stilestrongylus lanfrediae*	39838	Guarapuava - PR
Cestoda sp. 1	39840	Guarapuava - PR
***Euryoryzomys russatus***		
*Stilestrongylus eta*	39844	Balsa Nova - PR
*Stilestrongylus rolandoi*	39845	Balsa Nova – PR
Cestoda	39843	Balsa Nova - PR
***Mus musculus***		
*Stilestrongylus eta*	39846	Guarapuava - PR
*Stilestrongylus freitasi*	39848	Guarapuava – PR
*Syphacia evaginata*	39847	Guarapuava – PR
*Syphacia* sp. 5	39849	Guarapuava - PR
***Oligoryzomys nigripes***		
*Guerrerostrongylus zeta*	39850	Balsa Nova - PR
*Pterygodermatites* sp.	39851	Balsa Nova – PR
*Stilestrongylus lanfrediae*	39852	Balsa Nova – PR
*Syphacia carlitosi*	39853	Balsa Nova – PR
*Syphacia evaginata*	39854	Balsa Nova – PR
*Syphacia* sp. 1	39855	Balsa Nova – PR
*Guerrerostrongylus zeta*	39856	Guarapuava - PR
*Syphacia* sp. 3	39857	Guarapuava - PR
